# A single factor explanation for associative learning performance on colour discrimination problems in common pheasants (*Phasianus colchicus*)

**DOI:** 10.1016/j.intell.2018.07.001

**Published:** 2019

**Authors:** Jayden O. van Horik, Ellis J.G. Langley, Mark A. Whiteside, Joah R. Madden

**Affiliations:** Centre for Research in Animal Behaviour, Psychology, University of Exeter, UK

**Keywords:** General intelligence, Associative learning, Reversal learning

## Abstract

It remains unclear whether performance of non-human animals on cognitive test batteries can be explained by domain general cognitive processes, as is found in humans. The persistence of this dispute is likely to stem from a lack of clarity of the psychological or neural processes involved. One broadly accepted cognitive process, that may predict performance in a range of psychometric tasks, is associative learning. We therefore investigated intra-individual performances on tasks that incorporate processes of associative learning, by assessing the speed of acquisition and reversal learning in up to 187 pheasants (*Phasianus colchicus*) on four related binary colour discrimination tasks. We found a strong, positive significant bivariate relationship between an individual's acquisition and reversal learning performances on one cue set. Weak, positive significant bivariate relationships were also found between an individual's performance on pairs of reversal tasks and between the acquisition and reversal performances on different cue sets. A single factor, robust to parallel analysis, explained 36% of variation in performance across tasks. Inter-individual variation could not be explained by differential prior experience, age, sex or body condition. We propose that a single factor explanation, which we call ‘*a*’, summarises the covariance among scores obtained from these visual discrimination tasks, as they all assess capacities for associative learning. We argue that ‘a’ may represent an underlying cognitive ability exhibited by an individual, which manifests across a variety of tasks requiring associative processes.

## Highlights

• Acquisition and reversal learning was assessed using binary colour discrimination tasks involving novel colour pairs.
• A single factor, robust to parallel analysis, explained 36% of variation in performance across tasks.
• Inter-individual variation could not be explained by differential prior experience, age, sex or body condition.
• We show that performance on associatively learned tasks can manifest in single factor explanations of cognitive ability.

## 1 Introduction

In both humans and non-human animals, an individual's performance on a battery of diverse psychometric tasks may be summarised by a single factor that typically accounts for ~40% of their variance, which has been considered to reflect domain general intelligence ‘*g*' (Burkart, Schubiger, & van Schaik, 2016; Deary, 2001). Although first reported in humans (Spearman, 1904), single factors have more recently been extracted for a range of species including non-human great apes (Hopkins, Russell, & Schaeffer, 2014), and other primate species (Banerjee et al., 2009; Herndon, Moss, Rosene, & Killiany, 1997), rodents (Anderson, 1993; Galsworthy et al., 2005; Galsworthy, Paya-Cano, Monleon, & Plomin, 2002; Kolata, Light, Grossman, Hale, & Matzel, 2007; Kolata, Light, & Matzel, 2008; Light et al., 2010a; Light, Grossman, Kolata, Wass, & Matzel, 2011; Locurto, Benoit, Crowley, & Miele, 2006; Matzel et al., 2003, Matzel et al., 2006), dogs (Arden & Adams, 2016) and birds (Isden, Panayi, Dingle, & Madden, 2013; Keagy, Savard, & Borgia, 2011; Shaw, Boogert, Clayton, & Burns, 2015).

The factor labelled as ‘*g*' (within species) is considered a useful index of intelligence, as it provides a measure of covariance in performance among disparate cognitive tasks (Nisbett et al., 2012; Spearman, 1904). Although there may be inter-individual neural (Duncan et al., 2000) or brain region (Haier, Jung, Yeo, Head, & Alkire, 2004) correlates of general intelligence performance in humans (Roth & Dicke, 2005), or correlates of brain (region) size with ‘*G*' (terminology for general intelligence across species) (Deaner, Van Schaik, & Johnson, 2006; but see Amici, Barney, Johnson, Call, & Aureli, 2012), it remains unclear how independent cognitive processes, such as associative learning performance, memory and executive function, contribute to this ability. One, potentially fruitful approach towards furthering our understanding of domain general processes may be to investigate consistency in intra-individual performance on batteries of tasks that are clearly underpinned by somewhat general, yet broadly recognised, cognitive processes. For example, in human psychometric test batteries, such as the commonly used Wechsler Adult Intelligence Scale III, particular clusters of tasks are specifically included to probe broad processes such as verbal comprehension, processing speed or working memory, which it is suggested are in turn governed by an over-arching ‘*g*' (Wechsler, 1997). One common, well established, cognitive process likely to underpin an individual's performance across a range of tasks for both human and non-human animals is associative learning – by which an individual forms a mental connection between representations of two stimuli (Shettleworth, 2010, pp. 105). This process is exhibited across taxa and is the subject of numerous well developed psychometric tests.

Associative learning ability can be assessed using acquisition and reversal learning tasks. In these tasks, an individual initially learns to discriminate between two cues, associating one with a reward and the other with a cost (acquisition). After learning these associations the reward contingencies are reversed and the previously unrewarded cue becomes rewarded (reversal). While both acquisition and reversal learning involve processes of associative learning, they are also associated with different neural mechanisms and brain regions (Bouton, 2004; Dalley, Cardinal, & Robbins, 2004; Eisenhardt & Menzel, 2007; Rygula, Walker, Clarke, Robbins, & Roberts, 2010). Yet despite the apparent simplicity of this process, high inter-individual differences in performance on these tasks are often reported. Accordingly, learning speed on acquisition and reversal discriminations, in a range of taxa, have been found to be influenced by age (Forster et al., 1996), early life conditions (Brust, Krüger, Naguib, & Krause, 2014), motivation (Behmer, Belt, & Shapiro, 2005), personality (Guenther, Brust, Dersen, & Trillmich, 2014; Guillette, Reddon, Hoeschele, & Sturdy, 2011; Guillette, Reddon, Hurd, & Sturdy, 2009), ecological experience (Federspiel et al., 2017) and sex (Lucon-Xiccato & Bisazza, 2016). While the aforementioned factors can influence learning speed, most studies of single factor explanations of cognitive performance in animals (with the exception of some studies of rodents) differ from those in humans, in that they include subjects that differ greatly in age, prior experience or motivation. When this is coupled with relatively small sample sizes (N typically < 100), performances resulting in high single factor loadings may instead be caused by inter-individual differences in non-cognitive traits, such as prior experience, rather than an underlying cognitive ability. Finally, in many tests on non-human animals, particularly those presented to subjects in the wild, only single exemplars of each task are presented. Hence, it remains unclear whether an individual's performance is consistent and accurately reflects their cognitive ability, independent of the particular test paradigm or cue set deployed (Rowe & Healy, 2014).

We used an appetitively motivated acquisition and reversal learning paradigm to investigate whether associative learning performance, across multiple tasks, could be explained by a single factor. If this single factor represents a substantial amount of individual variation in task performance, we may therefore consider that a common process, namely associative learning, underlies both acquisition and reversal learning performance. Our subjects, Common Pheasants (*Phasianus colchicus*), hereafter pheasants, were tested at the same age and under controlled conditions. Individual experiences from birth prior to testing were standardised and we tested a relatively large sample of both males and females. This procedure therefore allowed us to also explore whether body condition (mass/tarsus^3^), sex or an interaction between the two may explain individual differences in performance. To ensure that performances could be attributed to the same underlying cognitive process, we replicated each task with novel sets of colour cues.

## 2 Methods

### 2.1 Subjects

We reared 200 day-old pheasant chicks in groups of 50 in four replicated enclosures between 28 May 2015 and 29 July 2015. All subjects were individually marked using numbered wing tags, fed on commercial pheasant feed supplemented with wild bird seed (~5%) and supplied with water ad libitum*.* Birds were housed in 2 m × 2 m heated huts for the first 2 weeks of life. They had access to unheated but covered outdoor runs of 1 m × 4 m for the next week and for the final seven weeks of rearing had access to 4 m × 12 m outdoor runs. All birds were tested with a battery of psychometric tests (including those detailed in this study) from 10 days old, with equal exposure in a fixed order to all tasks (van Horik, Langley, Whiteside, & Madden, 2017). During test sessions subjects could enter the experimental chamber (75 cm × 75 cm) at will, where they were tested individually while visually isolated from other test subjects. Morphometrics (mass, tarsus length) were taken and sex confirmed by plumage features at ten weeks old when testing ceased.

### 2.2 Procedures

Subjects were initially trained, using shaping procedures, to peck through a layer of crepe paper and retrieve a mealworm reward concealed in a well (van Horik et al., 2017). During testing, subjects were presented with two appetitively motivated colour discrimination tasks (Green/Blue 28 June – 01 July 2015; Yellow/Pink 06–10 July 2015) involving an acquisition learning phase and a reversal learning phase. Each task required subjects to discriminate between two wells in which the contents were concealed by a layer of crepe paper. One well contained a mealworm reward while the other well was made inaccessible by covering it with hard black card placed under the crepe paper, which could not be pecked through. Rather than leaving the well empty, we considered the black card to provide a more salient cue of an incorrect choice. Each well was encircled by one of two colour cues. During the Green/Blue acquisition phase, the rewarded well was associated with a green cue and the unrewarded well was associated with a blue cue. In the Green/Blue reversal phase the previously rewarded green well was no longer rewarded and the previously unrewarded blue well became rewarded. During the Yellow/Pink acquisition phase the rewarded well was associated with a yellow cue and the unrewarded well associated with a pink cue, and vice versa during the reversal phase.

Each subject was presented with five sessions in the acquisition phase and five sessions in the reversal phase. Subjects received two sessions per day, one in the morning and one in the afternoon, making ten binary choices (hereafter ‘trials’) in each session. Therefore, each bird received a total of 50 trials in the acquisition phase and 50 trials in the reversal phase. Birds experienced their first 10 reversal trials on the same day as their last 10 acquisition trials, but in the afternoon test session. A correct choice was scored if subjects first pecked into a rewarded well and an incorrect choice was scored if subjects first pecked into an unrewarded well. If the bird made a correct choice, it was allowed to eat the reward. If the bird made a wrong choice, the pair of wells was removed and replaced with a new pair. The location of the rewarded well was pseudorandomised across trials, and did not occur on the same side for more than three consecutive trials. Rather than standardising each individuals level of learning, by training to criterion, we provided a fixed number of trials on the acquisition and reversal discriminations. Hence, like Raine and Chittka (2012), we assessed individual performances on the different cue sets at the population level, by standardising each individual's opportunities to learn.

### 2.3 Fitting individual learning curves

We used learning curves to summarize individual performance across trials. Four learning curves were generated for each individual, one for each of the four different tasks. Learning curves were generated for 187 individuals that completed all 50 trials in at least one of the four discrimination tasks. However, our analyses across the different Acquisition and Reversal tasks were restricted to only 111 individuals that completed all trials on all four tasks (*n* = 59 males, *n* = 49 females, and three individuals for which we did not have sex or body condition measures). The coefficients describing learning curves were derived from the probability of whether or not a given subject made a correct or incorrect choice per trial, after fitting a sigmoid curve to the binary choice data using R (R Development Core Team, 2014). For our learning criteria, we used the predicted trial number when the curve exceeded an 80% probability of the bird making a correct choice. We derived this measure by solving the eq. *X* = (−ln0.25 – *b*_*0*_)/*b*_*1*_, where *b*_*1*_ is the slope of the learning curve, and *b*_*0*_ is the intercept. Learning curves accounted for individuals with a strong positive bias, as these birds showed poor improvement in performance. Our derived trial numbers were log transformed prior to analysis to improve normality of the data.

### 2.4 Sex and body condition

At 10 weeks old, after testing had ceased, all subjects were visually sexed and their body condition (mass/tarsus3) was determined by measuring each individuals mass using a spring balance scale (Slater Super Samsom – precision 5 g) and tarsus length using a calliper (precision 0.1 mm).

### 2.5 Statistical analysis

All statistical analyses were conducted in SPSS v23 (IBM Corp, 2013). We used paired *t*-tests to assess improvement in performance across sessions at the population level, by comparing the proportion of correct choices in the first 10-trial session to those of the final 10-trial session made by all birds. Repeated measures ANOVA were used to test whether the number of total correct choices differed with task and cue set. Binomial tests (set at 0.5) were used to assess whether subjects' first choices (correct or incorrect) were biased towards a particular colour cue.

Performance measures generated from learning curves from birds that completed all 200 trials on all four discrimination tasks were assessed using Principal Axis Factoring (PAF). We used the first unrotated component, to determine whether individual performances across all four tasks could be explained by a single factor. Rather than using Kaisers K1 criterion of including eigenvalues >1, which has been considered an inaccurate and arbitrary validation (Fabrigar, Wegener, MacCallum, & Strahan, 1999), we used Parallel Analysis to assess the likelihood that the eigenvalues generated from the PAF differed significantly from chance, following O'Connor (2000). To do this we ran 1000 randomised permutations, generated from the raw data set, and compared the raw mean eigenvalues from the PAF with the 95th percentile permuted eigenvalues. One-tailed tests were used to assess bivariate correlations, generated from the PAF Correlation Matrix following Field (2005), as we predicted an individual's performance in one colour discrimination task would correlate positively with their performance in a similar, albeit novel, colour discrimination task. Overall measures of sampling adequacy were assessed using Kaiser-Meyer-OlkinFs (KMO) tests and considered satisfactory if >0.5. Sampling adequacy for each task were also assessed using the anti-image correlation matrix and considered satisfactory if KMO > 0.5. Bartlett's test for sphericity was used to determine whether correlations between variables included in the inter-correlation matrix were acceptable (*P* < .05). Determinant scores were used to assess multi-collinearity and were considered adequate if >0.00001. To determine the model fit, residual differences between the observed correlation coefficients and the reproduced correlation coefficients generated from the factor model were compared. Models with <50% of bivariate residuals >0.05 were considered adequate (Field, 2005). Factor loadings for tasks >0.4 were considered salient (Stevens, 2002). To determine whether performances on the discrimination tasks were influences by non-cognitive explanations associated with appetitive motivational differences, we ran separate General Linear Models (GLM) to determine whether sex and body condition (mass/tarsus^3^) influenced learning curve performance on each task. When tests of sphericity were not met, we used lower bound estimates. All *P* values ≤ 0.05 were considered significant.

### 2.6 Ethical note

All work was conducted under Home Office licence PPL 30/3204 and approved by the University of Exeter Animal Welfare Ethical Review Board. Birds were habituated to human observation from one day old. Shaping procedures, using meal-worm rewards, were adopted to habituate subjects to the testing arena. These procedures were considered to mitigate stress and encouraged subjects' voluntarily participation during testing. There were no enforced aversive stimuli. Birds that failed to engage with the task in <2 mins were permitted to pass into the recovery area and their lack of participation recorded. Birds were reared at a lower density than that recommended by DEFRA's code of practice (DEFRA, 2009), thus reducing likely stress and competition between chicks.

## 3 Results

### 3.1 Do subjects' performances improve across sessions?

**Fig. 1:**
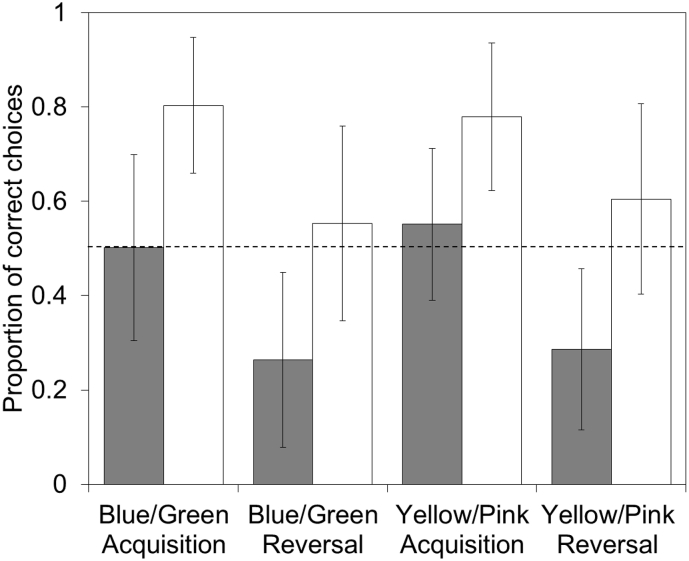
Differences in the mean proportion of correct choices made by individuals in their first (grey bars) and last (white bars) set of ten choices in a set of acquisition and reversal tasks with binary choices based on two sets of colour cues. The dashed line indicates 50% random choice. Error bars indicate ±1SD.

At a population level, birds learned to associate particular colour cues with rewards in acquisition and reversal tasks, across two different sets of colour cues (Table 1, Fig. 1). In the acquisition tasks, subjects increased their probability of a correct choice from chance levels of around 0.5 to around 0.8 after 50 trials. In the reversal tasks, subjects started with an incorrect preference for the previously rewarded colour, resulting in a probability of correct choices of around 0.27, which increased to a probability of around 0.57 after 50 trials. While subjects improved their performances during reversal trials, they made fewer correct choices on the reversal conditions compared to acquisition conditions (Repeated Measures ANOVA: F_1,156_ = 358.97, *P* < .001; Acquisition = 32.3 ± 0.44, Reversal = 20.1 ± 0.45), indicating that reversal learning performances were impaired by the previous associations learned during the acquisition phase. Subjects showed improved performances in the Yellow/Pink discriminations, compared to the Green/Blue discriminations (Repeated Measures ANOVA): F_1,156_ = 8.764, *P* = .004. First choices were compared for all birds on each task (Binomial test; Table 2). There was a bias at the population level towards the unrewarded colour (Blue) on the first Acquisition task, and a bias towards the rewarded colour (Yellow) on the second Acquisition task. Both Reversal tasks also showed a bias towards the previously rewarded colour.

**Table 1:** Differences in the proportions of correct choices between the first (1−10) and last (41–50) ten choices in a set of acquisition and reversal tasks with binary choices based on two sets of colour cues.

Task	*Paired T-test*	*P*
Green/Blue Acquisition	*t*_*174*_ *= 16.8*	<0.001
Green/Blue Reversal	*t*_*176*_ *= 17.9*	<0.001
Yellow/Pink Acquisition	*t*_*171*_ *= 13.1*	<0.001
Yellow/Pink Reversal	*t*_*174*_ *= 16.6*	<0.001

**Table 2:** A comparison (using binomial tests set at 0.5) of the first choices, correct and incorrect, that were made by each subject for the Acquisition and Reversal tasks for each of the colour cue sets.

	Green/Blue	Yellow/Pink
Acquisition	Correct = 61 Incorrect = 105 *P* = .001	Correct = 105 Incorrect = 74 *P* = .025
Reversal	Correct = 25 Incorrect = 159 *P* < .001	Correct = 20 Incorrect = 165 *P* ≤ .001

### 3.2 Are individual learning performances consistent across tasks?

Significant positive bivariate relationships were revealed in three of the six correlations between tasks (Table 3). While all other variables included in the inter-correlation matrix showed weak bivariate relationships, they remained acceptable (Bartlett's test for Sphericity *P* = .011).

**Table 3:** Correlation Matrix, generated from PAF, with associated *P* values (Sig. 1-tailed). KMO tests of sampling adequacy for each variable are presented in square brackets on task diagonals.

	Green/Blue Acquisition	Green/Blue Reversal	Yellow/Pink Acquisition	Yellow/Pink Reversal
Green/Blue Acquisition	[0.569]	0.285 P = .001	0.062 *P* = .258	0.193 *P* = .021
Green/Blue Reversal		[0.568]	0.116 *P* = .112	0.157 *P* = .05
Yellow/Pink Acquisition			[0.519]	−0.037 *P* = .349
Yellow/Pink Reversal				[0.600]

**Fig. 2:**
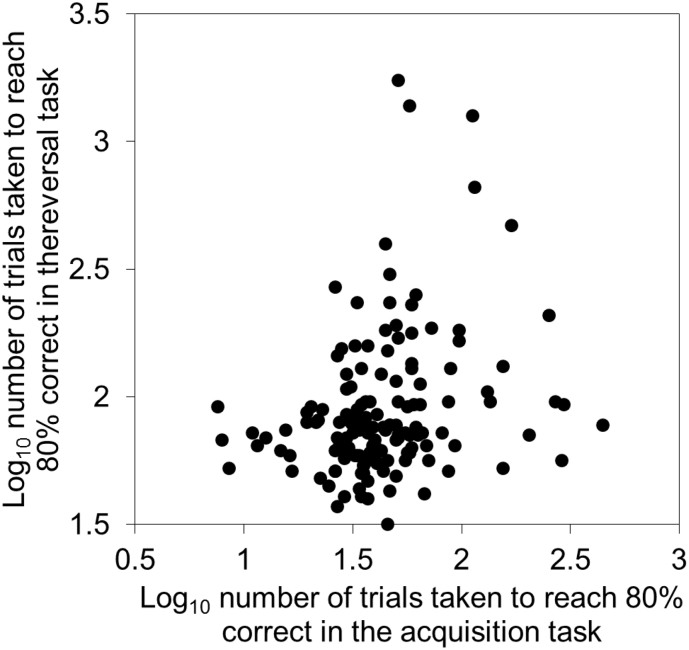
The relationship between the speeds of learning of an individual in an acquisition task and its reversal based on the discrimination of blue and green cues. Speed of learning is measured by the number of trials taken to reach a probability of being 80% correct, derived from learning curves. (For interpretation of the references to colour in this figure legend, the reader is referred to the web version of this article.)

**Fig. 3:**
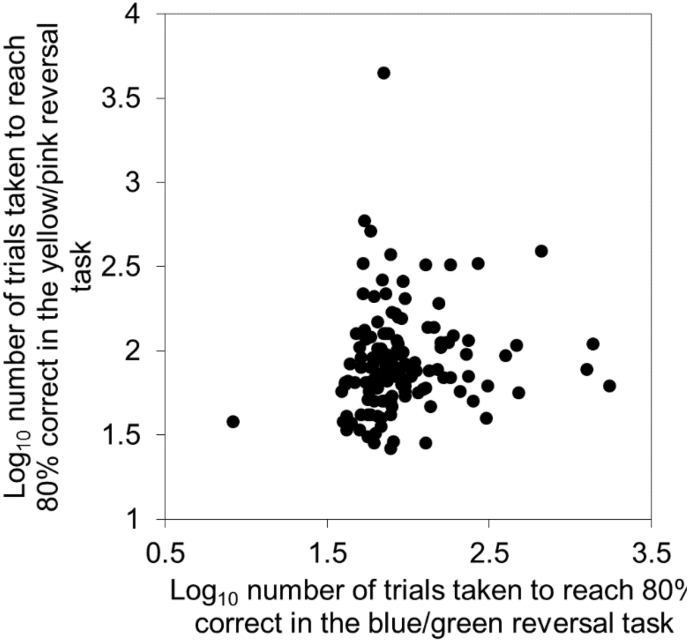
The relationship between the speeds of reversal learning, measured by the number of trials taken to reach a probability of being 80% correct, derived from learning curves, by an individual in two reversal tasks using different sets of colour cues.

**Fig. 4:**
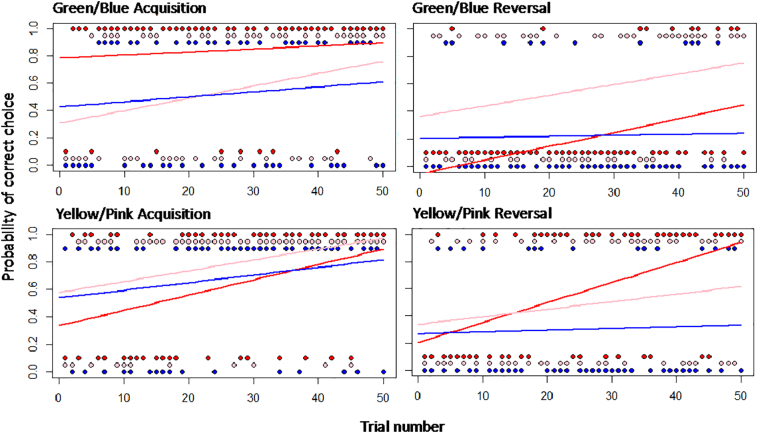
Individual performances across trials for each binary discrimination task. Performances are generated from learning curves that were derived from the probability of an individual making a correct or incorrect choice per trial. Individuals depicted are those with the highest (red line: bird 279), median (pink line: bird 384), and lowest (blue line: bird 441) single factor scores generated from PAF of their aggregate performances. (For interpretation of the references to colour in this figure legend, the reader is referred to the web version of this article.)

Although individuals that rapidly acquired an association between a colour and a reward were also fast to reverse that same association in the Green/Blue cue set (Fig. 2), this relationship was weak and not significant in the Yellow/Pink cue set (Table 3). Individuals' that were fast learners in one reversal task were also fast in the other reversal task (Table 3; Fig. 3, relationships remain when outliers removed). However, performances on one of the acquisition tasks did not relate to performances on the other acquisition task (Table 3). Green/Blue Acquisition correlated significantly with Yellow/Pink Reversal, although Yellow/Pink Acquisition did not correlate significantly with Green/Blue Reversal (Table 3). Single factor scores, generated from PAF of aggregate performances, were used to determine birds with the highest (bird 279), median (bird 384), and lowest (bird 441) overall performances. The performances of these individuals in each task are illustrated in Fig. 4.

### 3.3 Can learning performances be explained by a single factor?

The inter-correlation matrix did not suffer from multi-collinearity (Determinant = 0.858) and the overall sample size was adequate (Kaiser-Meyer-Olkin test = 0.571), as was the sample size for all individual tasks (Table 3; presented in square brackets on task diagonals). Only two (33%) bivariate residuals generated from reproduced correlation coefficients were > 0.05 (0.051 and 0.075), suggesting that the coefficients derived from the factor model show an excellent fit to the observed correlations (i.e. < 50%; Field, 2005).

A single factor accounted for 36.18% of an individuals' variation in performance across all four tasks. This value was a higher than expected by chance (Parallel analysis: 95% permuted Eigenvalue = 0.41, Actual Eigenvalue = 0.54). All other remaining eigenvalues were non-significant (Factor 2: 95% permuted Eigenvalue = 0.17, Actual Eigenvalue = 0.07).

All four variables loaded positively onto the first factor (Table 4). High factor loadings (> 0.4; Stevens, 2002) were revealed in two of the four tasks, and confined within the Green/Blue colour tasks (Table 4). Factor loadings were weak on the yellow/pink tasks.

**Table 4:** Factor matrix reporting loadings of the four colour discrimination tasks on the first factor, and mean ± Standard Deviation (SD) of Log_10_ predicted number of trials to reach 80% correct for each discrimination problem.

Task	Factor 1	Mean ± SD
Green/Blue Acquisition	0.561	1.62 ± 0.32
Green/Blue Reversal	0.525	1.93 ± 0.33
Yellow/Pink Acquisition	0.123	1.75 ± 0.41
Yellow/Pink Reversal	0.308	1.92 ± 0.31

### 3.4 Are acquisition or reversal learning performances influenced by sex or body condition?

None of the individual acquisition and reversal learning curve performances or the single factor scores generated from PAF varied with sex or body condition (Table 5).

**Table 5:** The effects of an individual's sex, body condition and the interaction between them on their learning speed in four binary discrimination tasks and a single factor that underlies their performance. *F* values are given for each GLM. Associated *P* values are given in parentheses.

Task		Sex	Condition	Sex*Condition
Green/Blue Acquisition	F_1,155_	0.26 (0.61)	0.26 (0.61)	0.28 (0.60)
Green/Blue Reversal	F_1,158_	0.17 (0.68)	0.02 (0.90)	0.19 (0.67)
Yellow/Pink Acquisition	F_1,140_	0.36 (0.55)	1.83 (0.18)	0.22 (0.64)
Yellow/Pink Reversal	F_1,157_	2.13 (0.15)	0.31 (0.58)	2.08 (0.15)
Factor 1	F_1,108_	0.43 (0.52)	0.53 (0.88)	0.81 (0.54)

## 4 Discussion

Pheasant chicks learned to associate colour cues with food rewards, showing improvements in their performances across all four tasks. When reward contingencies were reversed, pheasant chicks also demonstrated the ability to learn to inhibit their previously learned responses. Individuals showed variation in their rates at which they learned and reversed these associations. Performances across these appetitively motivated colour discrimination tasks could also be explained by a single factor, suggesting that some individuals may be overall faster learners than others.

Individuals that were fast to acquire an association were also fast to inhibit these previously learned contingencies, both within the Green/Blue acquisition and reversal colour set and across the Green/Blue acquisition and Yellow/Pink reversal colour sets. Although, at the population level, success on the reversal tasks only reached approximately 60% after 50 trials, subject's performances improved from an initial success of approximately 30% correct choices. Hence, while their chance performance suggests they failed to learn the reversed associations, such an improvement in performance suggests that subjects learned to inhibit responding to the previously learned contingencies. Accordingly, subjects' impaired performances on the reversal discriminations and their subsequent improvement reflect capacities for inhibition learning (Izquierdo & Jentsch, 2012). Acquisition and reversal learning performance has previously been found to correlate positively in song sparrows and bumblebees (Boogert, Anderson, Peters, Searcy, & Nowicki, 2011; Raine & Chittka, 2012). A positive relationship indicates that both processes may be governed, at least to some extent, by a shared underlying process, such as associative learning ability. Yet, our findings were inconsistent across subsequent, novel, colour pairings where no relationship was observed (Yellow/Pink). Future studies may therefore benefit from investigating these discrepancies by comparing individual consistency in discrimination performance on both similar, i.e. colour, and dissimilar, i.e. shape, cue sets (Griffin, Guillette, & Healy, 2015).

In contrast to our findings, poor acquisition learners have also shown superior reversal learning performances, for example in Florida scrub-jays (Bebus, Small, Jones, Elderbrock, & Schoech, 2016), Indian mynas (Griffin, Guez, Lermite, & Patience, 2013) and black-capped chickadees (Guillette et al., 2011). This may be because individuals that frequently sample unrewarded cues are more exploratory, which may contribute to their improved performance on reversals (Federspiel et al., 2017; Guillette, Hahn, Hoeschele, Przyslupski, & Sturdy, 2015). Studies that report a negative relationship between acquisition and reversal learning assessed subjects' performances by comparing the mean number of errors (Bebus et al., 2016; Guillette et al., 2011) or total number of trial blocks (Griffin, Lermite, Perea, & Guez, 2013) an individual made before reaching a predetermined criterion of success. Hence, studies reporting a negative relationship standardised each individual's level of learning before presenting them with a reversal problem. In the current study, we used similar procedures to Raine and Chittka (2012), and generated individual learning curves to assess subjects' performances across a fixed number of trials. We therefore standardised each individual's opportunity to learn each discrimination problem. These different techniques (training to criteria or the use of learning curves from fixed numbers of trials) may therefore explain the discrepancies between acquisition and reversal performance between these studies. However, in both techniques, individuals that were slower, or even failed, to learn the acquisition phase have greater experience with the unrewarded stimuli than those individuals that rapidly learn the associations. Therefore, in both cases slow acquisition learning may aid reversal performance. Yet, it remains that strong associations made during the acquisition phase should initially impair subjects' responses to the previously rewarded contingencies during the reversal phase. Although, some species may use strong acquisition learning criteria to generate rule-based responses and hence aid their reversal learning performance (Rumbaugh & Pate, 1984; van Horik & Emery, 2018).

An individual's performance on the reversal task for one cue set predicted (albeit weakly) their performance on the reversal, but not the acquisition phase of a novel cue set. It remains difficult to interpret why our subjects showed greater consistency in their reversal learning performance when compared to the first acquisition task, but not when compared with the second acquisition task. We can speculate that there may be more consistency in reversal learning performance in contrast to acquisition learning, because this involves another, separate cognitive process, i.e. inhibition. Given that different neural/molecular mechanisms or brain regions likely govern reversal performance compared to acquisition learning (Bouton, 2004; Dalley et al., 2004; Eisenhardt & Menzel, 2007; Rygula et al., 2010), associative processes may therefore be more strongly expressed or weighted in reversal learning tasks that require additional processes of inhibition. In contrast, in acquisition tasks cue salience is more influential, perhaps due to sensory biases. However, low repeatability in performance across different inhibitory control tasks has also been found in pheasants (van Horik et al., 2018).

Non-human animals tested in the wild are often presented with single task exemplars that are considered to reflect particular, domain specific, cognitive capacities (Isden et al., 2013; Keagy et al., 2011; Shaw et al., 2015). However, the assumption that a single task accurately captures or represents a domain specific cognitive capacity requires investigation. In our study with the exception of the Green/Blue acquisition and reversal tasks, between subjects' correlations across similar tasks were only weakly related; although these relationships remained significant in three of the six bivariate correlations. In several other studies that have searched for a single factor that accounts for an individuals general cogntivie ability, weak bivariate correlations have also been reported between different tasks. For example 8 of 15 (Shaw et al., 2015), 6 of 15 (Isden et al., 2013) and 12 of 15 (Keagy et al., 2011) tasks showed bivariate correlation coefficients of <0.25. While these other studies may be constrained by a small sample size (typically looking at <20 individuals), single factor explanations for cognitive performance persist, both within similar cognitive tasks and across different cognitive domains (Burkart, Schubiger, & van Schaik, 2017). Such findings contrast with those of human studies and from non-human apes tested in captivity which utilise larger sample sizes, and for which performances across multiple exemplars of similar tasks cluster within specific cognitive domains (Herrmann, Call, Hernandez-Lloreda, Hare, & Tomasello, 2007; Herrmann, Hernández-Lloreda, Call, Hare, & Tomasello, 2010; Wechsler, 1997). To further our understanding of the evolution of cognition, it therefore remains important to understand how an individual's performance on a task represents their abilities in a particular cognitive domain of interest (Griffin et al., 2015; Shaw & Schmelz, 2017).

Although the relationship between an individual pheasant's performance on appetitively motivated tasks of acquisition and reversal, both within and across the different colour pairs, were typically weak, they could be robustly summarised by a single factor. This factor explained ~36% of variation in inter-task performance. Hence, individuals that were fast at learning one task could be considered generally successful at learning the other colour discrimination tasks. Perhaps surprisingly, the explanatory power of our first factor (36%) is similar to that found in studies investigating whether a general cognitive ability underlies cognitive performances in other animals (summarised in Burkart et al., 2016; mean 34.2%, range 27–48%). However, in contrast to these studies, our tasks were not designed to assess disparate cognitive domains, but rather they summarised general performance on discrimination tasks that required a cognitive processes, namely associative learning. Such processes may be particularly influential to performances on cognitive test batteries in animals but less so for humans (van Horik & Lea, 2017).

An individual's learning performance, both on individual tasks and when summarised by a single factor, could not be explained by a suite of potential confounding non-cognitive factors. All individuals were tested on the same days, so age could not contribute to differences. All individuals had been reared under identical conditions and experienced identical sets of stimuli in matched orders, so prior experience could not explain differential performances. Indeed, the stimuli were selected knowing that such colours had never previously been seen by the birds. We also attempted to reduce motivational effects by withholding access to the preferred food reward (mealworm) at all times except during testing, and quantifying performances using a binary response variable (success/failure), rather than e.g. time taken to access a reward or number of attempts made to access the reward. Therefore, we measured the manner in which a focal individual interacts with the test, making ‘correct’ or ‘incorrect’ choices, with a change in these frequencies over time, rather than simply the speed or intensity which they interact with the test. This gives us a clearer indication of the cognitive rather than motivational factors controlling individual performance. While we could not account for intrinsic differences in motivation, driven by appetitive preferences or metabolic variation, any inter-individual differences in learning speed were not predicted by sex, body condition or an interaction between these two. While previous studies of “g” in non-human animals (particularly in birds) often use food rewards, it remains possible that performances on the different tasks in our study were driven by a general motivation to acquire mealworms, rather than any specific cognitive ability. We consider this unlikely, as performances improved across trials. However, future studies may benefit from including tasks in which performance is mediated by non-appetitive reinforcers, such as those adopted in rodent studies that involve navigating a Lashley III maze, escaping an undesirable location such as water, fear conditioning or passive avoidance (e.g. Light et al., 2010b). We controlled for differences in the underlying association between the cue and a reward outcome by using novel variants of the same task, that presumably involves the same cognitive processes to discriminate between different colours. Shaping procedures ensured that all birds had learnt the operant response prior to testing. Finally, we only included birds that participated in all trials, hence controlling for any differences in their experience or willingness to participate. We therefore consider that individual differences in learning speeds primarily reflected cognitive ability.

We excluded individuals that failed to participate in every trial from our analyses. It therefore remains possible that our findings are biased towards some characteristic which both influences the likelihood that an animal will engage in these cognitive tests and produces a correlation in test performance between the four tests. For example, exploratory behaviours in mice covary with their general learning ability (Matzel et al., 2006). We have previously found that participation (but critically not performance) on cognitive tasks can differ according to non-cognitive traits in pheasants (van Horik et al., 2017). Consequently, as subjects could self-select for inclusion in the tests, our analyses may be constrained by inaccurate measures of cognitive variation. However, if our analyses are based on restricted range of cognitive ability, our findings may also underestimate the amount of variance that this single factor accounts for. Hence, we may expect broader cognitive variation to provide stronger support for our findings than the present analysis suggests. We could have overcome this potential confound by ensuring participation by all individuals in all task. However, this could have had two equally detrimental effects. If we had literally forced individuals into the test chamber, we would likely have induced additional stress which can have detrimental influences on cognitive performance (Li et al., 2008), so confounding our results. Alternatively, if we had waited and allowed individuals to participate whenever they felt like it of their own volition, we would have negated our intended advantage that all birds included in the analysis were tested at exactly the same ages and after exactly the same prior experiences. This constriction does not apply to studies that collect their data in a much less controlled manner – for example testing primates in zoos at any time, over any duration, with no constraints on prior experiences.

If test performance informs us about underlying cognitive abilities, rather than the effects of motivational or perceptual processes, then we might expect a stronger relationship in individual performances within a particular task type (acquisition or reversal) across different colour cue sets, than compared to their performances across tasks within a particular colour cue set. For example, acquisition learning using Green cues should be an equivalent predictor of acquisition learning using Yellow cues. However, factor loadings for tasks utilising the Green/Blue cue set were stronger than those observed for tasks utilising the Yellow/Pink cue set (Table 4). This could be because of different saliances of Green/Blue or it could be due to an order effect: all subjects received the Green/Blue tasks prior to the Yellow/Pink tasks. As the tasks were similar, it is possible that subjects' performance on the second discrimination problem was facilitated by their previous experience on the first task. This positive transfer of learning across similar tasks may contribute to an inflated general factor, and consequently obscure the process that influence performance across tests. However, we consider it unlikely that an order effect facilitated a transfer of learning on the second colour pair. Pheasants in the current study received 50 trials on two different colour pairs. By contrast, similar studies that have used visual discriminations to assess positive transfer, such as those on pigeons for example involving matching to sample (same vs different), require training of hundreds of different stimuli over thousands of trials before subjects show positive transfer (Katz & Wright, 2006; Wright, Cook, Rivera, Sands, & Delius, 1988). The decision of whether to counterbalance stimuli sets is problematic in studies where individual differences, rather than population differences, are the subject of interest (Bell, 2013). We decided not to counterbalance, but instead present each individual with a standardised set of stimuli in a matched order so that we could control for variation in their experiences and directly compare learning performances between individuals. Consequently, these procedures do not allow us to determine whether performances on the second acquisition task were influenced by previous experiences on the first task, a bird's age at testing, or were due to the particular colour cues presented. At a population level, performances on the second Yellow/Pink acquisition task improved, albeit marginally, compared to performances on the initial Green/Blue acquisition task (Fig. 1). It is likely, however, that performances on the second acquisition task were facilitated by a bias towards the rewarded colour (yellow), whereas performances on the first task were biased towards the unrewarded colour (blue; Table 2). While subsequent studies may clarify these findings by counterbalancing the presentation order of colour cues across subjects, we consider such biases unlikely to hinder our interpretations of the results. We observed no bias in first session performances in either of the acquisition tasks. Moreover, our learning measures were derived from individual improvements in performances, accounting for rates of improvement (*b*_*1*_), and when performances started to improve (*b*_*0*_). Hence, learning curves accounted for individuals with a strong positive bias, that failed to improve. Accordingly, we consider it unlikely that population level biases influenced our individual learning measures.

We tested a relatively large sample of pheasants with two novel binary colour discriminations on two different tasks (Acquisition and Reversal) associated with different neural mechanisms and brain regions (Bouton, 2004; Dalley et al., 2004; Eisenhardt & Menzel, 2007; Rygula et al., 2010). All tasks utilised associative learning and we found that a subjects' performance on the four colour discrimination problems could be summarised by a single factor. We suggest that the existence of such a single factor may support the notion of a general associative learning ability (we propose this should be called ‘*a*’, as opposed to a general intelligence (‘*g*') (van Horik & Lea, 2017). Accordingly, ‘*a*’ may broadly underpin an individual's general cognitive performance across a variety of different problems that specifically require capacities to associate reliable cues with rewarded outcomes through trial-and-error experience. As the unobservable nature of latent variables that comprise “*g*” are an elusive target for selection (Arden & Zietsch, 2017), opportunities for associative learning may therefore provide a platform for selection to act on (Dukas & Bernays, 2000; Dukas & Duan, 2000); with faster learning bringing fitness benefits (Raine & Chittka, 2008). Future studies may validate the notion of ‘*a*’, by investigating performances on other learning tasks, across modalities, and in different contexts. This may be done, for example, by associating visual cues with food palatability, predator detection, mate discrimination or opponent quality.

## Competing interests

The authors declare no competing interests.
